# Design, construction and field testing of a manually feeding semiautomatic sugarcane bud chipper

**DOI:** 10.1038/s41598-024-54980-3

**Published:** 2024-03-04

**Authors:** Abdallah Elshawadfy Elwakeel, Saher M. A. Mohamed, Abubakr Abdelwahab Tantawy, Abdelaziz M. Okasha, Salah Elsayed, Osama Elsherbiny, Aitazaz A. Farooque, Zaher Mundher Yaseen

**Affiliations:** 1 Agricultural Engineering Department, Faculty of Agriculture and Natural Resources, Aswan University, Aswan, Egypt; 2 General Department, Faculty of Agriculture, Cairo University, Giza, Egypt; 3 Agronomy Department, Faculty of Agriculture, Minia University, Minya, Egypt; 4 Department of Agricultural Engineering, Faculty of Agriculture, Kafrelsheikh University, Kafr El-Sheikh, 33516 Egypt; 5 Agricultural Engineering, Evaluation of Natural Studies and Research Institute, University of Sadat City, Sadat City, 32897 Minufiya Egypt; 6 New Era and Development in Civil Engineering Research Group, Scientific Research Center, Al-Ayen University, Nasiriyah, Thi-Qar 64001 Iraq; 7 Agricultural Engineering Department, Faculty of Agriculture, Mansoura University, Mansoura, 35516 Egypt; 8 Faculty of Sustainable Design Engineering, University of Prince Edward Island, Charlottetown, PE C1A4P3 Canada; 9 Canadian Centre for Climate Change and Adaptation, University of Prince Edward Island, St Peters Bay, PE Canada; 10 Civil and Environmental Engineering Department, King Fahd University of Petroleum & Minerals, 31261 Dhahran, Saudi Arabia; 11 Interdisciplinary Research Center for Membranes and Water Security, King Fahd University of Petroleum & Minerals, 31261 Dhahran, Saudi Arabia

**Keywords:** Semiautomatic sugarcane chipper, Sugarcane bud, Damage index, Invisible losses, Physical properties, Environmental monitoring, Civil engineering, Field trials, Design, synthesis and processing

## Abstract

Sugarcane is the main sugar crop, and sugar is an important agricultural product in Egypt. There are many problems with the technology used in the current planting method of sugarcane, which has a great impact on the planting quality of sugarcane, which have a series of problems, such as low cutting efficiency and poor quality. Therefore, the aim of the current study was to design, construct, and field testing of a semiautomatic sugarcane bud chipper assisted with pivot knives for cutting sugarcane buds and germinating them in plastic trays inside a greenhouse until they reached an average length of 35 cm, and then planting them in the field. In the field tests five cutting speeds (35, 40, 45, 50, and 56 rpm. (Revolution Per minute), three cutting knives (1.5, 2.0, and 2.5 mm) were used for cutting sugarcane stalks with four different diameters (1.32, 1.82, 2.43, and 2.68 cm). The obtained results showed that the values of the damage index and invisible losses were within acceptable limits (ranging between − 1.0 and 0.0) for all the variables under the test. Still, the lowest damage index and invisible losses were recorded with the buds that were cut with a knife of 1.5 mm thickness and cutting speeds less than 50 rpm. The skipping rate increases with the increase in cutting speed and stalk diameter, ranging between 0.0 to 13%. The maximum machine productivity was 110 Buds per minute at a cutting speed of 35 rpm and stalk diameter of 1.32 cm. The paper's findings have important application values for promoting the designing and development of sugarcane bud chipper and sugarcane planting technology in the future.

## Introduction

Sugarcane is the most important sugar crop, as it represents 80% of the global production of sugar, and it is also used in the production of biofuels and some types of renewable energies^1^. The sugarcane business contributes to economic growth and increases the income of the farmers. One tendency in industrial growth is the complete mechanization of the sugarcane process. Accordingly, it is very essential to pay full attention to sugarcane crop, to increase sugar productivity and decrease the gap between local production and the growing demand^2–4^.

Planting uniformity of sugarcane is a key evaluation index for planters^5^ and the cultivation by seedlings aims to save water, use fewer fertilizers, and raise the productivity of feddan in order to achieve a large income for the farms as well as a significant increase in sugarcane production, reaching 1.4 million tons of sugar annually within 3–5 years. The absence of a system for approving the seeds used in agriculture and ensuring their quality and that they are free from pests and diseases caused a decrease in the rates of germination of buds, which led to a decrease in plant density and the spread of pests and diseases, and thus a decrease in productivity. In addition to that, in traditional agriculture, each acre needs 6 tons of Sugarcane Stalk (SS), while in the cultivation of sugarcane by seedlings, it is sufficient to plant an acre with 1.5 tons of SS, which means saving about 10.7 tons of SS when planting a one hectare with seedlings. In addition to the presence of wide distances between seedlings and planting lines, which leads to the ease of following a modern irrigation system (drip irrigation), there is also the adoption of modern agricultural mechanization technology, which saves large amounts in weed control and reduces the number of manual hoeing times to only one as it allows the use of automatic hoeing. At a lower cost than manual harrowing^6^.

The sugarcane bud is very sensitive to damage, and the challenge is to separate it without any damage. Because any small scratch can cause damage to the buds and they will not germinate properly, which leads to spoilage^7^. Conventional hand-held sugarcane bud cutting instruments put stress on the hands and thumb, waste material, injure plants with slanting cuts, and cannot handle difficult plant grafting. This demands the development of a sugarcane bud cutting machine^8,9^.

The cutting force required for cutting sugarcane stalk, depends on the physio-mechanical characteristics of the SS and the knife thickness, and the force required to cut SS vary according to the cut position of the bottom, middle, or top of the stalk. Also, the cutting forces increase with the increase in the diameter of the stalks^10–12^.

Sugarcane C9 that was cultivated in Egypt had a mean diameter of 2.4 cm, a hardness of 775 N, a weight of 825 g, and a cutting force of 863 N^3,13^. A rotary cutting device with blades is the most suitable system for cutting thicker stalks (such as sugarcane), which have greater cutting resistance^14^. A key element influencing the amount of cutting force and power needed is the form of the cutting blade^15^. Compared to smooth knives, serrated knives had improved cut quality and cutting power, but the invisible loss was higher^3,16–18^. Liu et al.^16^, and Srivastava et al.^19^ investigated that the pressure of the cutting device initially causes a permanent deformation in the SS, which depends on the time of contact, knife thickness, and characteristics of the cutting device, which causes a break of the SS fiber. Neves et al.^20^ stated that invisible losses are representative of the cutting device that occur in all stages of harvester processing. Voltarelli et al.^21^ reported that the invisible losses in sugarcane affected by many reasons such as operator and cutting device.

Many researchers have designed, examined, and improved many Sugarcanes Bud Cutting Machine (SBCM) designs to date. Pujar et al.^22^ designed and tested a sugarcane bud chipping machine. Vertical reciprocating and double cutter types are included into the machine. The cutting mechanism was set to do 20 strokes per minute at a motor speed of 1330 rpm. The machine produced 30 buds every minute Elwakeel et al.^17^. Manufactured and assessed the effectiveness a machine for cutting sugarcane nodes. The cutting efficiency ranged from 83.67 to 100%, the machine output reached a maximum of 3944 buds per hour, and the total operating cost varied from 3.75 to 7.89 USD/hectare based on the cutting speed and stalk diameters. Ahmad et al.^23^ presented a sugarcane bud chip cutting machine prototype for nursery planting. The mechanical system comprised of a designed unit that was powered by compressed air supplied by a 10-bar air compressor. A pneumatic cylinder propels a specific punch that splits the cane stalk's buds. The physical and mechanical parameters of sugar cane buds cutting were measured. Under test settings, the machine produced an average of 1056 buds per hour. Jadhav et al.^7^ designed a semiautomatic sugarcane bud cutting machine. The machine is designed to avoid damaging the sugarcane bud while remaining efficient. One bud is cut every two seconds by the model. This is comparable to existing machines on the market, but at a lower cost. This gadget costs Rs. 20,360. A market survey indicates that a similar equipment costs 40,000 INR. The manufactured equipment produces 1800 buds every hour. Meeting the performance standards. Mahmoud and Abu El-maaty^24^ created a machine that sliced sugar cane buds. The machine was evaluated at three different transmission ratios to cutting rate: R1 = 22, R2 = 32, and R3 = 40 buds/min. Preliminary testing revealed that the machine obtained skipping, percentage of damaged, cutting efficiency, and productivity of (4.09, 7.19, 11%), (2.37, 5.39, and 8.99%), (97.63, 94.61, 91%), and (1266, 1782, 2136 bud/hr.) at the cutting rates R1, R2, and R3, respectively. The total operating costs of the machine are 50.3 L E/h at the highest cutting rate R3.

This study presented the design, construction, and field testing of a Semiautomatic Sugarcane Bud Chipper (SSBC) assisted with a novel cutting knives (pivot knives) for cutting and separation of sugarcane buds. To the end, a SSBC was designed according to standard methodology, where double pivot knives were constructed on the machine frame. For the SSBC validation, the effect of operation parameters (cutting speed, knife thickness and sugarcane stalk diameter) on machine productivity, invisible losses, machine productivity and skipping rate. The operation parameters were applied to find the optimum operation parameters for maximizing the machine productivity and minimize the damage index and invisible losses. Finally, the separated buds were planted in plastic trays inside a greenhouse until they reached an average length of 35 cm, and then planted in the field. There are many things considered in the design and construction of the SSBC, like light weight, the possibility of moving it from one place to another, safety, reliability, high productivity, manufacturing costs.

## Materials and methods

### Materials

**Figure 1:**
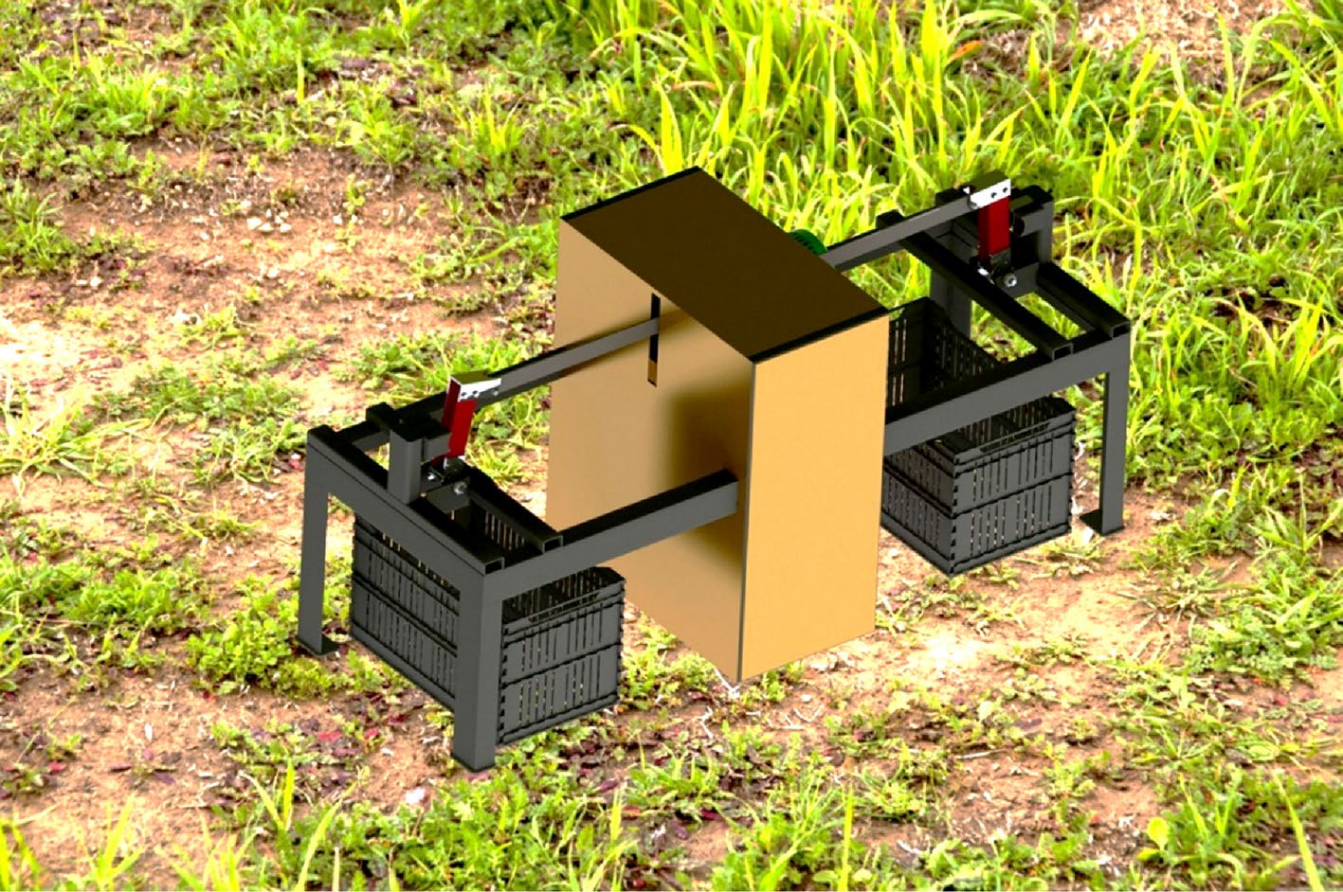
Isometric view of the SSBC.

For achieving the research aims, an SSBC was manufactured, developed, and constructed at a workshop in El-Minya governorate—Egypt, as shown in Fig. 1. All field experiments were carried out during season 2022 in the Research Farm at El-Minia University (RFMU).

#### Design and specifications of the machine elements

The SSBC was designed and developed based on standard methodology. The SSBC consists of three parts, as illustrated in the following figure.

##### Machine frame

**Figure 2:**
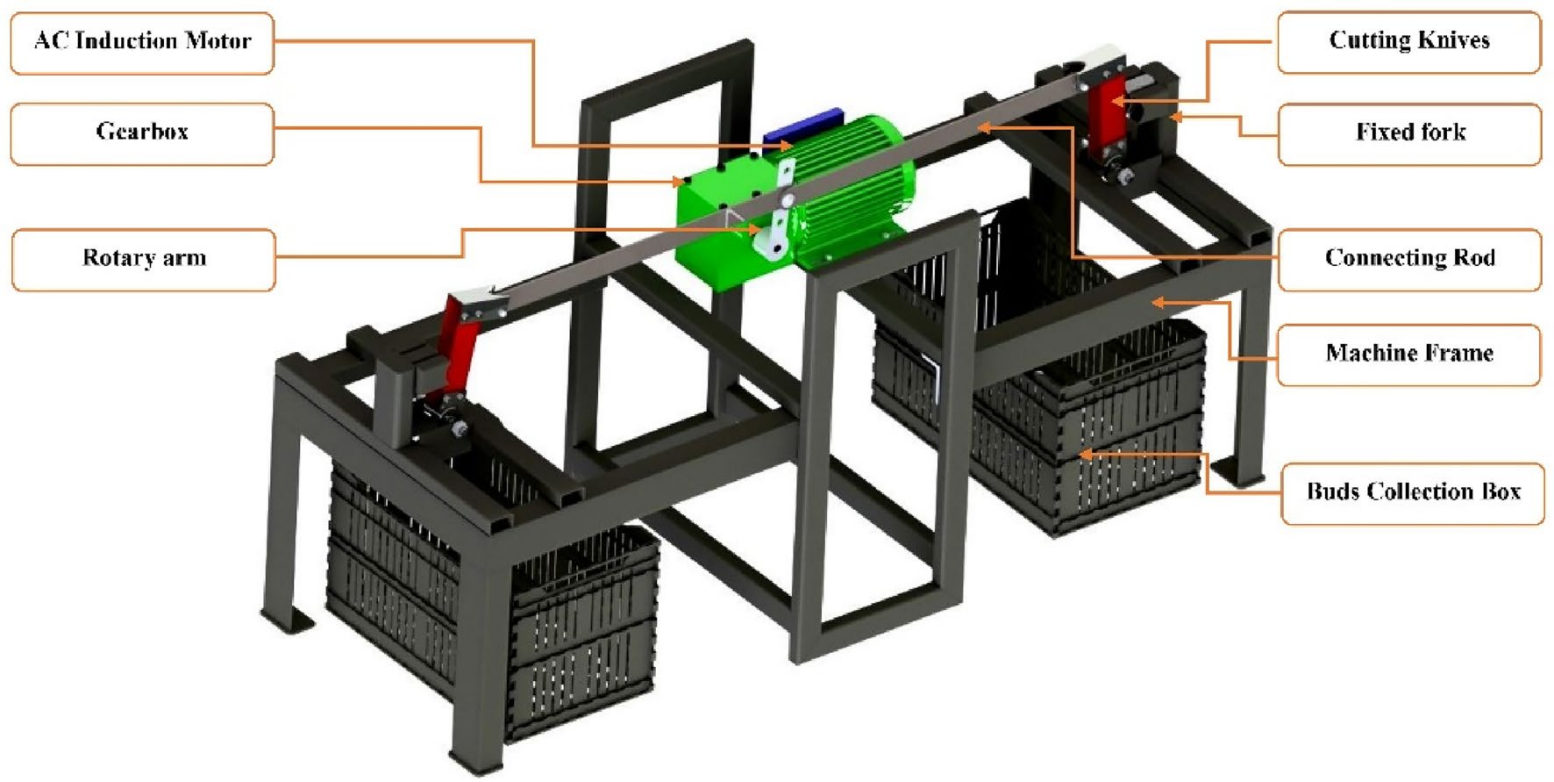
The different parts of the SSBC.

The frame of the SSBC carries all the components such as the cutting device, power source, and transmission system (gear box), as shown in Fig. 2., in addition to resisting the various forces and stresses that result from operating the SSBC or cutting the sugarcane stems. Some important points were considered in the design of the frame: lightweight, high resistance to loads and stresses, low manufacturing cost, reliability, availability of spare parts in local markets, and operator comfort during operation.

**Figure 3:**
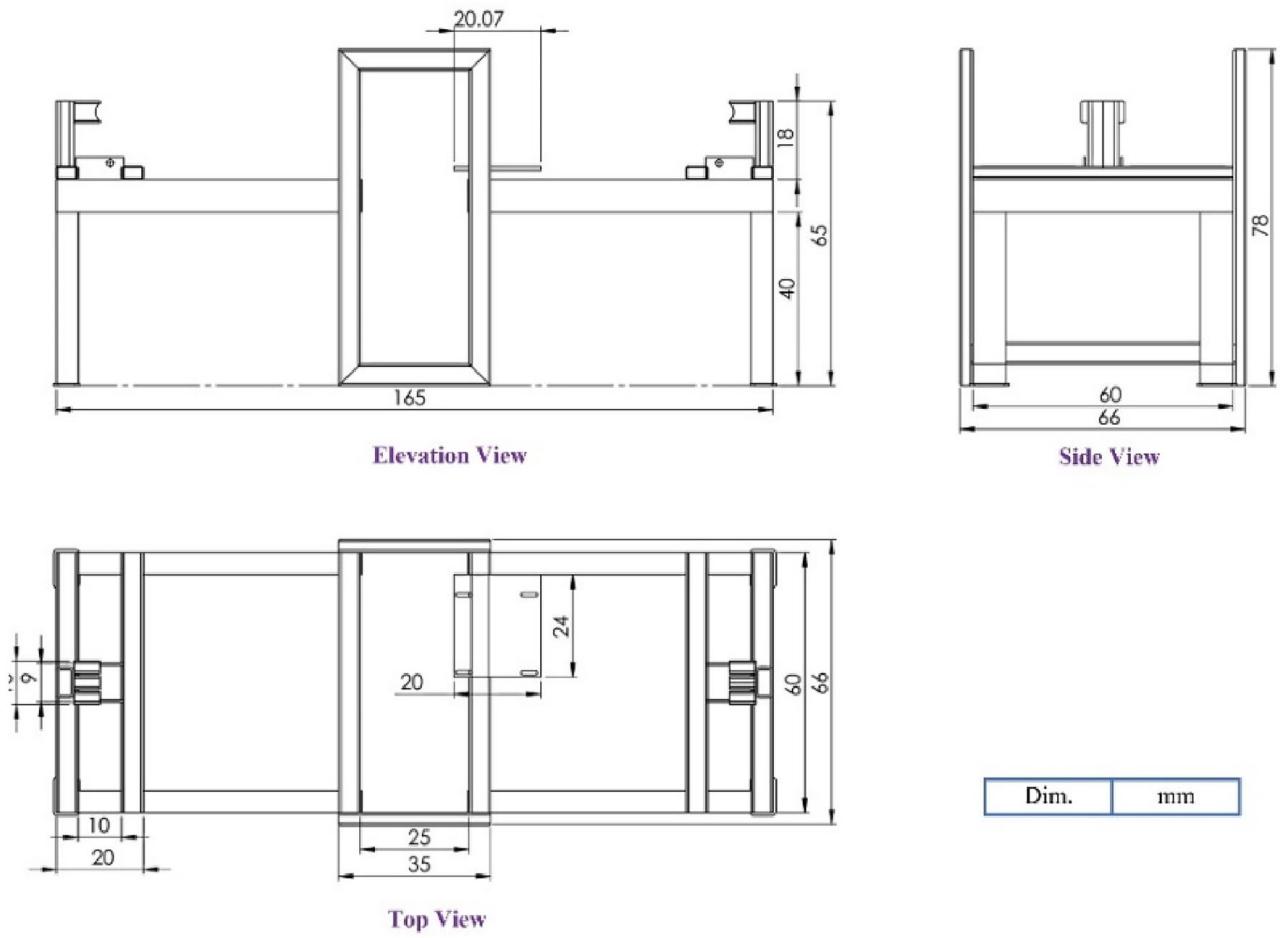
Detailed drawings of the SSBC.

The frame of the machine was manufactured from angle bars 3 × 2 × 1/4 in and rectangular tubes 76 × 38–1.6 mm & 40 × 20–2.0 mm—Mild Steel (hollow section). The main dimensions of the machine frame, as shown in Fig. 3. are 78 * 165 * 66 in height, length, and width, respectively, as well as the detailed drawings of the SSBC are shown in Fig. 3.

##### Cutting device

**Figure 4:**
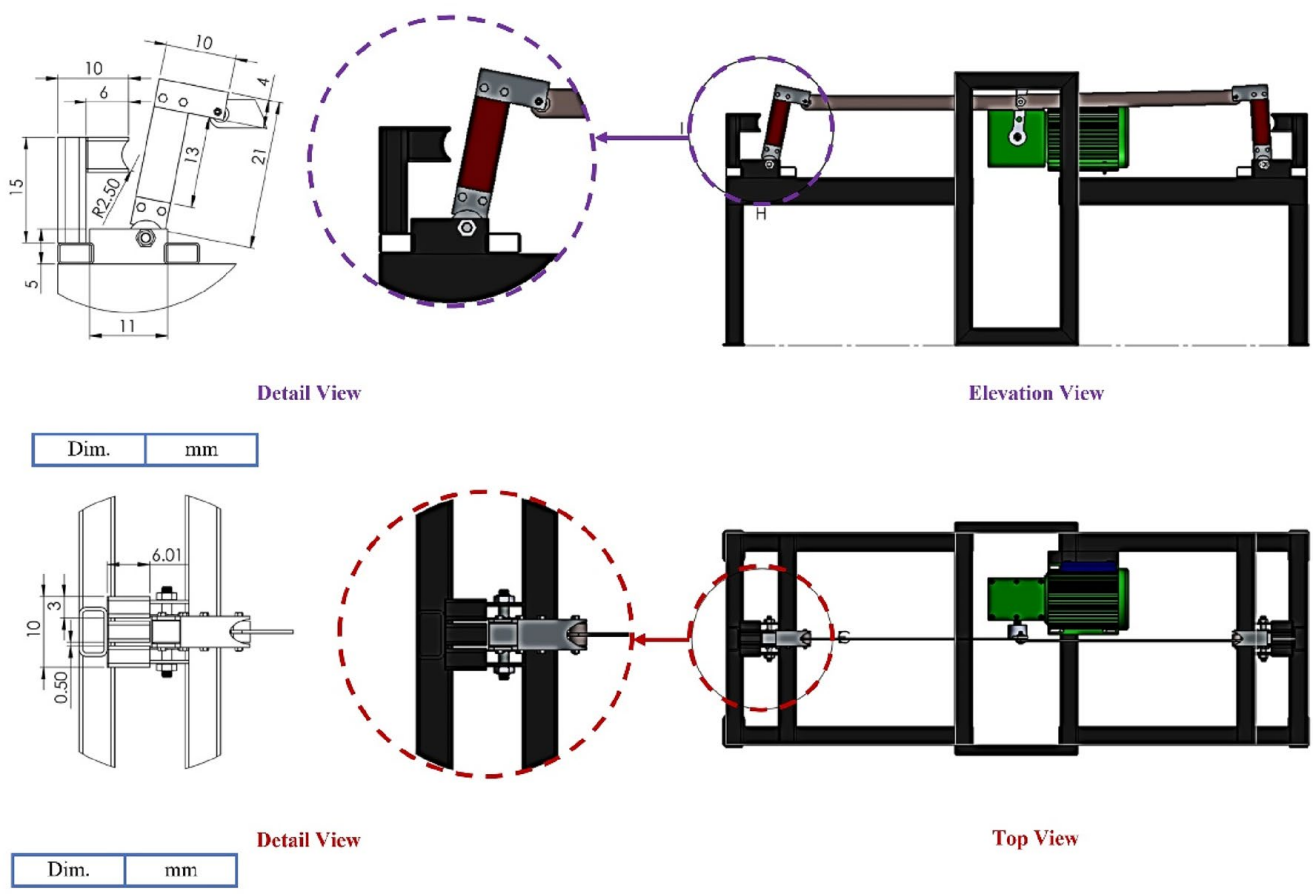
Detailed views of the cutting device and pivot knives.

As shown in Fig. 4, the cutting device consists of upper and lower forks, stainless steel smooth knives, connecting rods, pivot hubs, bolts, pins, and fixed forks. The upper and lower forks are made of iron plates with dimensions of 15 cm in height, 10 cm in width, as shown in Fig. 4. An iron plate with measurements of 64.2 cm in length, 30 mm in width, and 5 mm in thickness is used to make the connecting rod. The main purpose of the connecting rod is to make the joint connection between the electric motor and the cutting device, as shown in Fig. 4.

**Figure 5:**
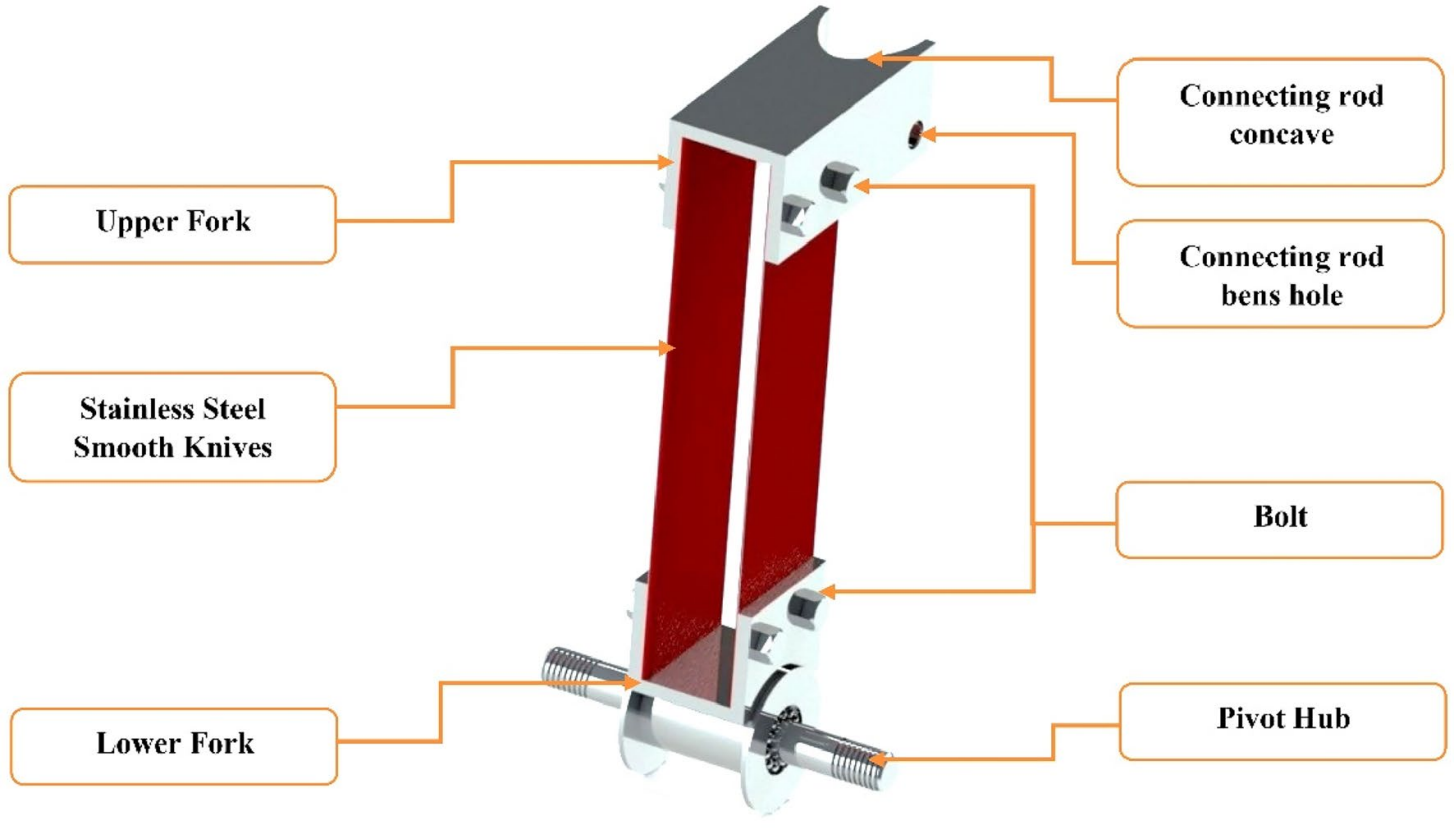
The main parts of the cutting device.

The SSBC contains two cutting devices, one for each side, and each cutting device has two knives. It was sharpened by the LASER. The distance between stainless steel knives is 3.5 cm depending on the desired length of buds, as shown in Fig. 5.

##### Power source

The SSBC is operated by a three-phase alternative current (induction motor) (model: WA30DT80KA/ASD1 (Germany)) of 3/4 hp (0.55 kW), rotational speed 360/56 rpm., and operating current 3.05–1.75 A. The operating speed was reduced from 360 to 56 rpm. by using a gearbox with an output torque of 93.3 Nm. The cutting speed was controlled using a dimmer (a voltage regulator device).

##### Adjustment and operation

**Figure 6:**
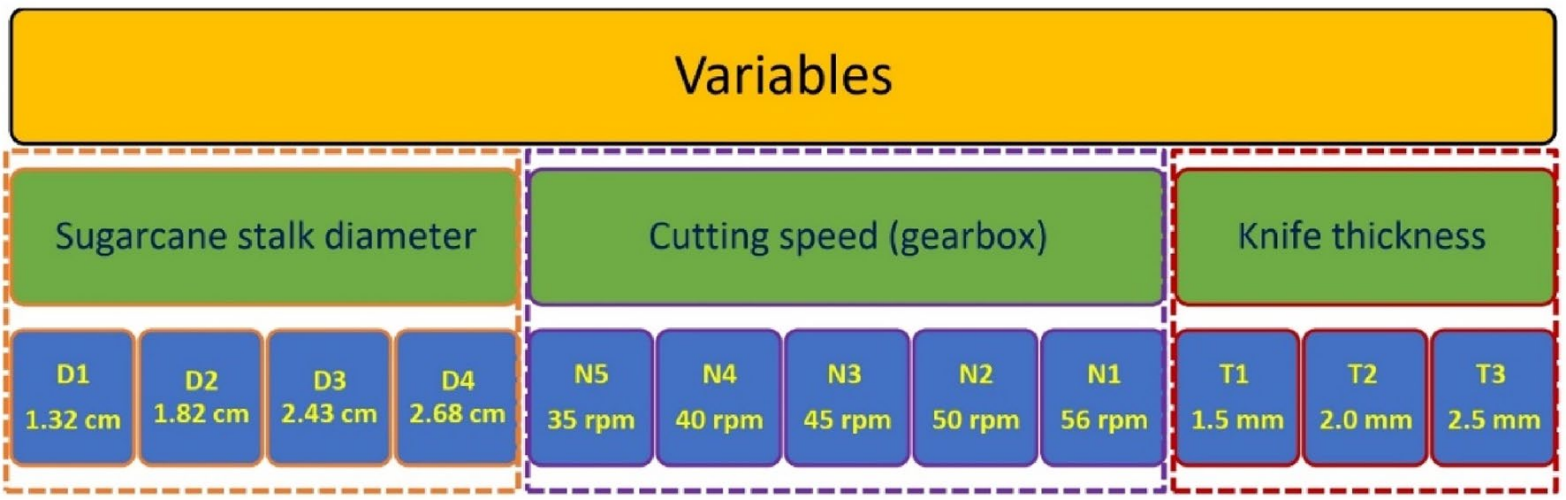
Flow chart showing the variables that are studied during the field tests of the SSBC.

**Figure 7:**
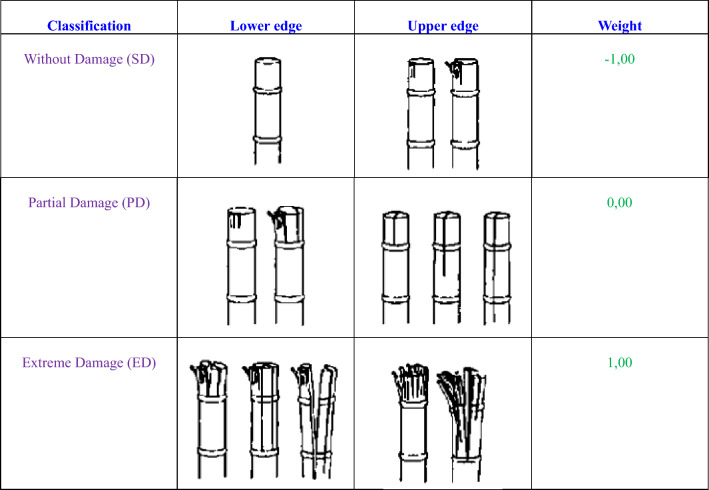
Classification of damage caused to SS, according to ^25,26^.

As shown in Fig. 6, in the beginning, the SS (commercial variety C9) are cleaned using hand knives, in order to make it easier to see the buds to be cut later. after that the operator holds the SS where the bud is located between the stainless-steel knives and the fixed fork. After separation, all buds fall into the collection box, and the internodes are still on the ground surface under the SSBC, as shown in Fig. 7. After that, the sugarcane buds are planted in plastic trays containing the previously prepared soil mixture in a greenhouse in September 2022, in the RFMU. The left-over sugarcane (internodes) can be used for preparing sugarcane juice, sugar, or jaggery.

### Methods

The SSBC was tested and evaluated in the RFMU, where field experiments were conducted to estimate:

- Some physical properties of SS are under test.
- SSBC performance in terms of:
- Damage index.
- Invisible losses.
- Machine productivity.
- Skipping rate.

#### Design of the field study

Field testing was carried out to test and assess how well the SSBC performed while looking at the variables shown in Fig. 6.

#### Measurements

##### Machine productivity ($Q$)

The productivity of the SSBC was calculated according to^8,17^. As the SSBC was operated by an experienced worker, both number of buds cut and consumed time per whole SS were recorded, and then the SSBC productivity (bud/min) was calculated using Eq. 1.

1 $$Q = \frac{{Nb}_{act}}{t}$$

##### Skipping rate (Sr)

Skipping occurs due to a number of reasons, the most important of which is the increase in the rotational speed of the cutting knives beyond the feeding rate that can be matched by the operator, and the skipping rate can be calculated based on Eq. 2, according to^24^.

2 $$Sr= \frac{{Nb}_{Theo}- {Nb}_{act}}{{Nb}_{Theo}} \times 100$$

##### Damage index (ID)

To determine buds' quality as a function of SS diameter, cutting speed, and knife thickness, four different SS diameters (D1 = 1.32, D2 = 1.82, D3 = 2.43, and D4 = 2.8 cm) for the three cutting knives (t1 = 1.5, t2 = 2.0, and t3 = 2.5 mm) were subjected to five cutting speeds (N1 = 35, N2 = 40, N3 = 45, N4 = 50, and N5 = 56 r.p.m., and repeated three times at least. The cutting buds in the collecting box were then individually analyzed and classified according to the guidelines that reported by^25,26^ as shown in Fig. 7.

Each classification represents a weight used for the calculation of the ID according to the equation developed by^26^, and Filho et al.^27^. The ID represents a way of converting qualitative aspects into quantitative or numerical ones. All analyses were performed by the same evaluator for greater statistical control, and the ID was calculated using Eqs. (3) and (4).

3 $$ID= \frac{{P}_{SD}.{n}_{SD}+ {P}_{PD}.{n}_{PD}+ {P}_{ED}.{n}_{ED} }{n}$$

4 $${n= n}_{SD}+ {n}_{PD}+{n}_{ED}$$

##### Invisible losses (${L}_{i}$)

The invisible losses during bud cutting were calculated according to Eq. (5) adapted from Filho et al.^27^, and Neves et al.^20^, The difference between the weight of the SS before cutting, and after cutting represents the invisible losses.

5 $${L}_{i}= {W}_{i}- \left[\left[{w}_{n}\times {N}_{n}\right]+\left[{w}_{i}\times {N}_{i}\right]\right]$$

##### Statistical analysis

The obtained data were analyzed, using descriptive statistics. All statistical tests were performed using the SPSS software 25.

## Results and discussion

### Physical characteristics

The sugarcane samples used in the field tests were harvested from El-Minya Governorate. A commercial cultivar, C9, was used in the field tests of the SSBC. Immediately after the harvesting process, the SS were transported to RFMU to be cleaned manually in preparation for the process of cutting the buds.

**Figure 8:**
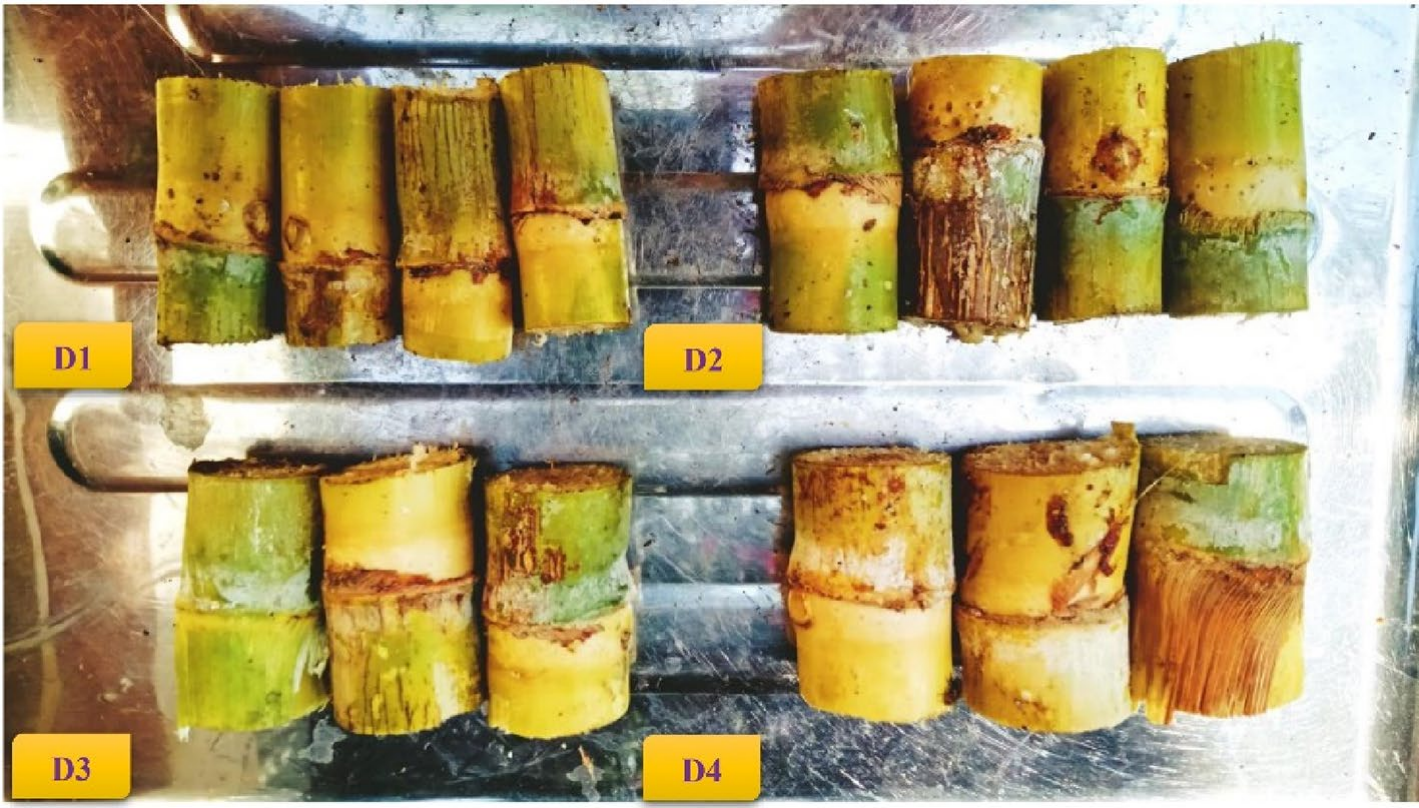
The four groups of SS samples based on stalk diameter.

The clean SS were classified into four parts according to the mean diameter of the SS, and the mean diameters in the four groups were (D1 = 1.32, D2 = 1.82, D3 = 2.43, and D4 = 2.68 cm), as shown in the Fig. 8. The mean diameter (top, middle, and bottom), mean weight, and mean length of the SS were shown in Table 1.

**Table 1:** Some physical characteristics of SS.

Sample No	SS diameter, cm				Mean weight, g	Mean length, cm
	Bud position on the SS					
	Bottom	Middle	Top	Mean		
D1	1.45 ± 0.12	1.30 ± 0.07	1.22 ± 0.06	1.32 ± 0.08	506.32 ± 65	175.15 ± 7.29
D2	2.03 ± 0.08	1.79 ± 0.05	1.63 ± 0.08	1.82 ± 0.07	790.18 ± 95	190.34 ± 8.37
D3	2.64 ± 0.07	2.41 ± 0.09	2.24 ± 0.10	2.43 ± 0.09	989.36 ± 98	182.37 ± 4.38
D4	3.06 ± 0.09	2.87 ± 0.11	2.65 ± 0.08	2.68 ± 0.09	1097.81 ± 154	190.91 ± 5.27

± Standard division.

### Damage index (DI)

**Figure 9:**
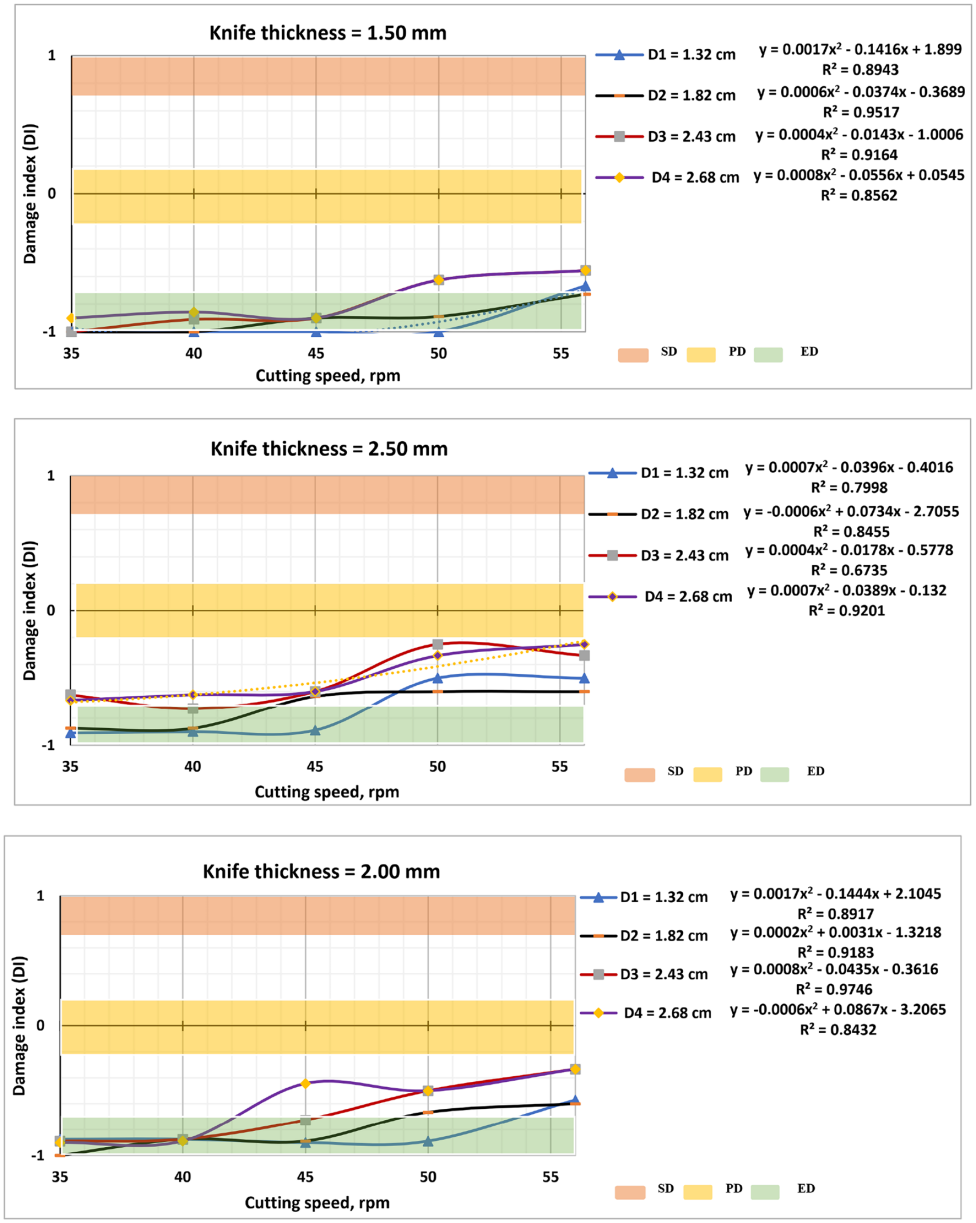
The relationship between ID and the cutting speed.

The obtained results of DI as a function of the cutting speed (N), mean diameter (D), and knife thickness (t), are shown in Fig. 9. DI values were less than 0.00 for all thicknesses of cutting knives, cutting speeds and stalk diameters under testing. This indicating a good cut quality of the cutting knives relative to the cutting systems, with results similar to those obtained by^25,28,29^, where^28^, stated that the DI value for straight blades of -0.6, corroborating the results obtained in this work. DI values remained below 0.00, defined as partial damage, which indicates that the cutting devices are acceptable. However, this may be because they are new cutting tools, with few hours of use as stated by Filho et al.^27^.

Figure 9 shows the relationship between ID and the cutting speed. Where it was found that the highest values of DI were recorded at a cutting speed equal to 35 rpm. and mean diameter is 2.68 cm. DI values decreased by increasing the cutting speed until it reached the lowest value of 56 rpm. Filho et al.^27^ stated that for longer exposure durations, the cutting knives caused more damage than shorter exposure durations.

**Figure 10:**
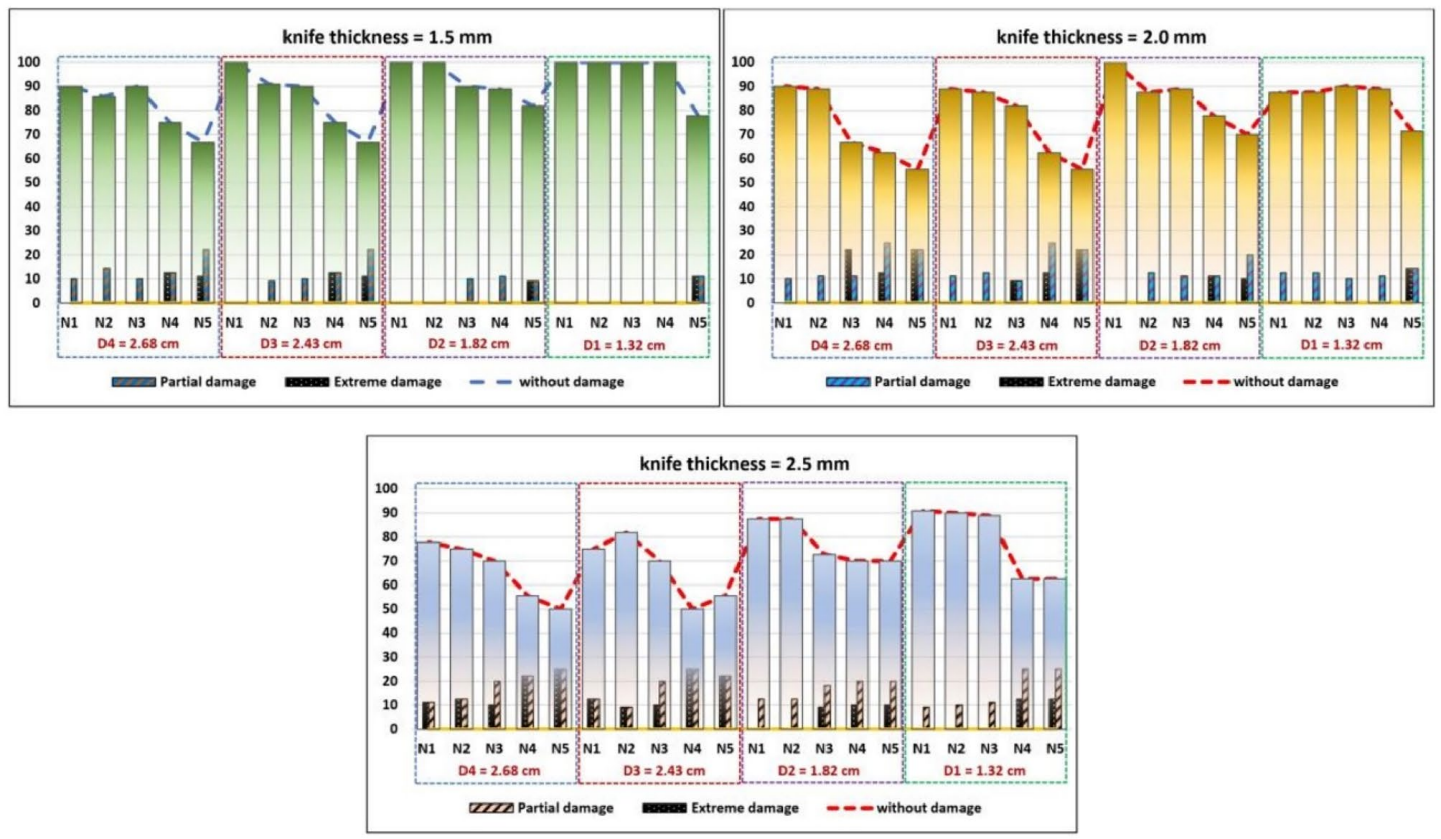
Frequency of damage as a function of cutting speeds.

The highest and lowest values of DI were recorded at mean diameter 2.8, and 1.32 cm, respectively. Also, the use of cutting knives in 2.5 mm thickness resulted in greater damage compared to the other knives, as shown in Fig. 10.

The damage frequency of the sugarcane buds, expressed as a percentage, is shown in Fig. 10. At a cutting speed of 35 to 50 rpm, a knife thickness of 1.5 mm, and an average stalk diameter of 1.32 cm, no partial or extreme damage was recorded in all cutting buds, and the percentage of buds without damage was 100%, which indicated the optimal performance of the cutting device. The percentage of separated buds without damage increased, while the percentage of buds cut with partial damage and extreme damage decreased by increasing the cutting speed. At the same cutting speed, knife thickness, and mean diameter of 2.68 cm, the extreme damage (partial damage) percentage was 13% (13%) and 11% (22%) at 50 and 56 rpm, respectively. As shown in the same figure, we find that using cutting knives with a thickness of 2.0 and 2.5 mm leads to an increase in partial damage and extreme damage in the separated cuttings, and the same thing happens when the stalk diameter is increased. Accordingly, the highest percentage of damage recorded during all tests was when using a cutting knife with a thickness of 2.5 mm, a stalk diameter of 2.68 cm, and a 35 cutting speed. Filho et al.^27^ reported that the extreme damage is reduced by the lower contact times with the cutting device compared to the high speed.

It was found that the SS may have been pushed during the displacement process, which resulted in splits and lacerations during the cut and doubled the incidence of serious damage^30^. While cutting a vegetable, the fibers are squeezed forward and to the sides of the cutting tool, and consecutive rupture processes occur as the tool advances^19^, which confirms the variability of the results in this study. The percentage of damaged stalks is similar to those obtained by^31^ in a field study, where the damage percentage was inversely proportional to the cutting speed.

### Invisible losses (Li)

**Figure 11:**
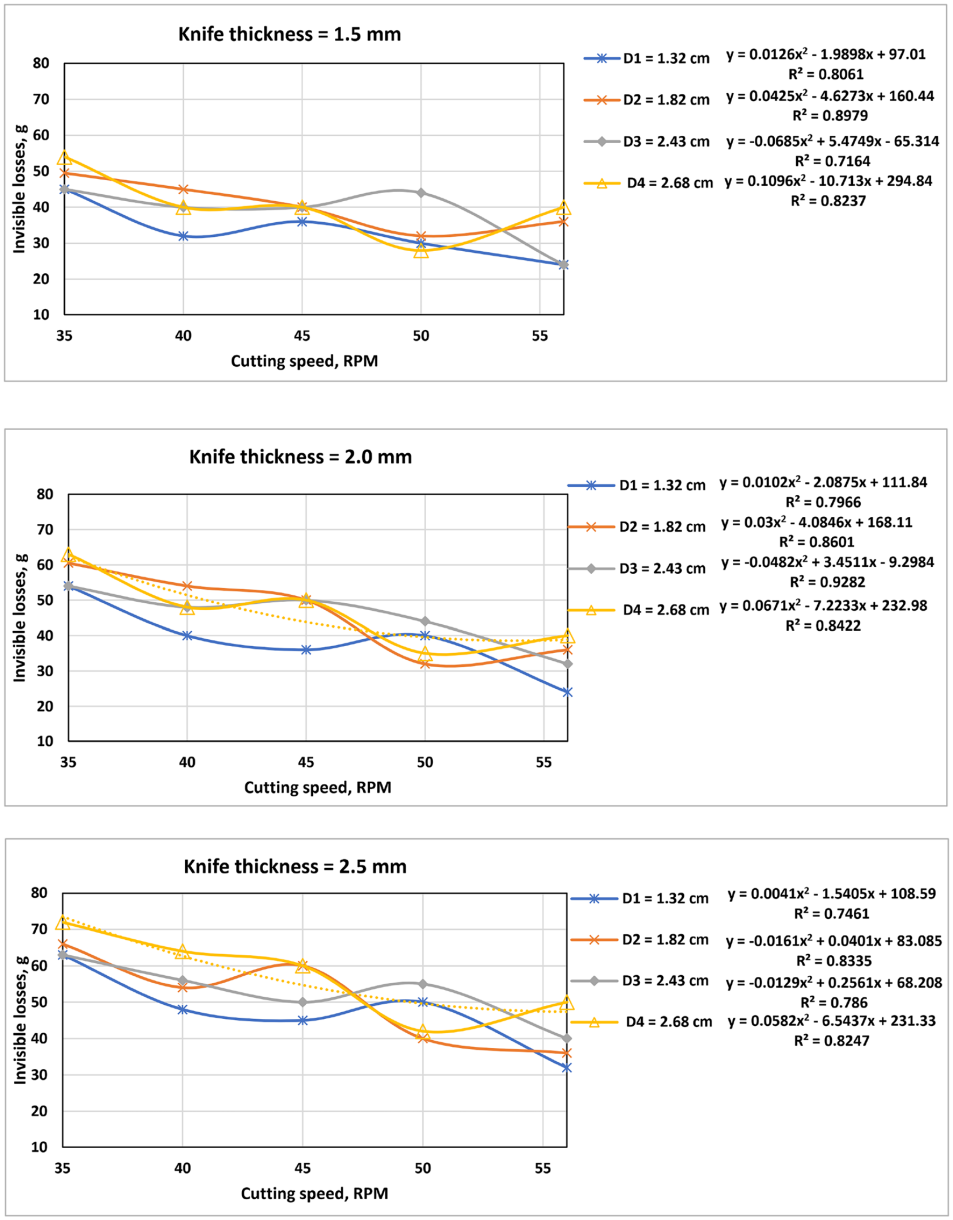
Invisible losses a function of cutting speed.

The cutting knives thicknesses of 2.0 and 2.5 mm had a maximum invisible loss of 63 and 72 g, respectively, at a cutting speed of 35 rpm and an average stalk diameter of 2.8 cm. The average weight of the SS used in this study was 1097.8 ± 154 g. Thus, maximum invisible loss per each SS ranged from 5.65 to 6.56%. The minimum invisible losses were recorded for the cutting knife thickness of 1.5 mm at a cutting speed of 56 rpm and an average stalk diameter of 1.32 cm as shown in Fig. 11.

### Skipping rate

**Figure 12:**
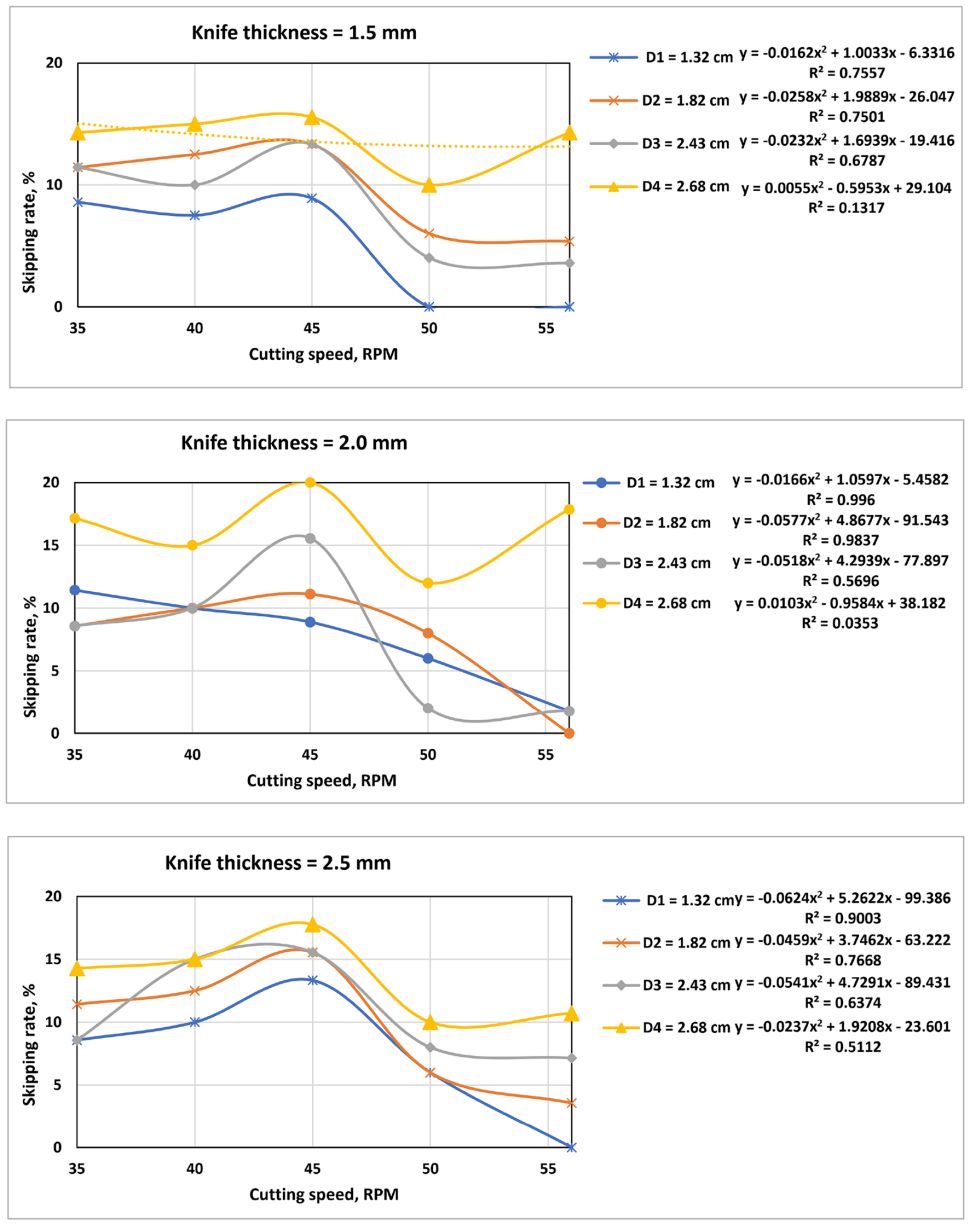
Skipping rate as a function of cutting speed.

The skipping rate is one of the most important factors that determine the productivity of the machine. Through it, it is possible to determine the best speed of the cutting system that is commensurate with the feeding speed of the operator to obtain the best productivity of the SSBC. The skipping rate is due to the increased reciprocating speed of the cutting device. As shown in Fig. 12. It can be seen that the relationship between the skipping rate and the cutting speed and the stalk diameter is inversely related, as the skipping rate increases with the increase in the cutting speed and the stalk diameter, and this is due to the operator's inability to feed at a rate commensurate with the cutting speed, as according^24^.

### Machine productivity (MP)

**Figure 13:**
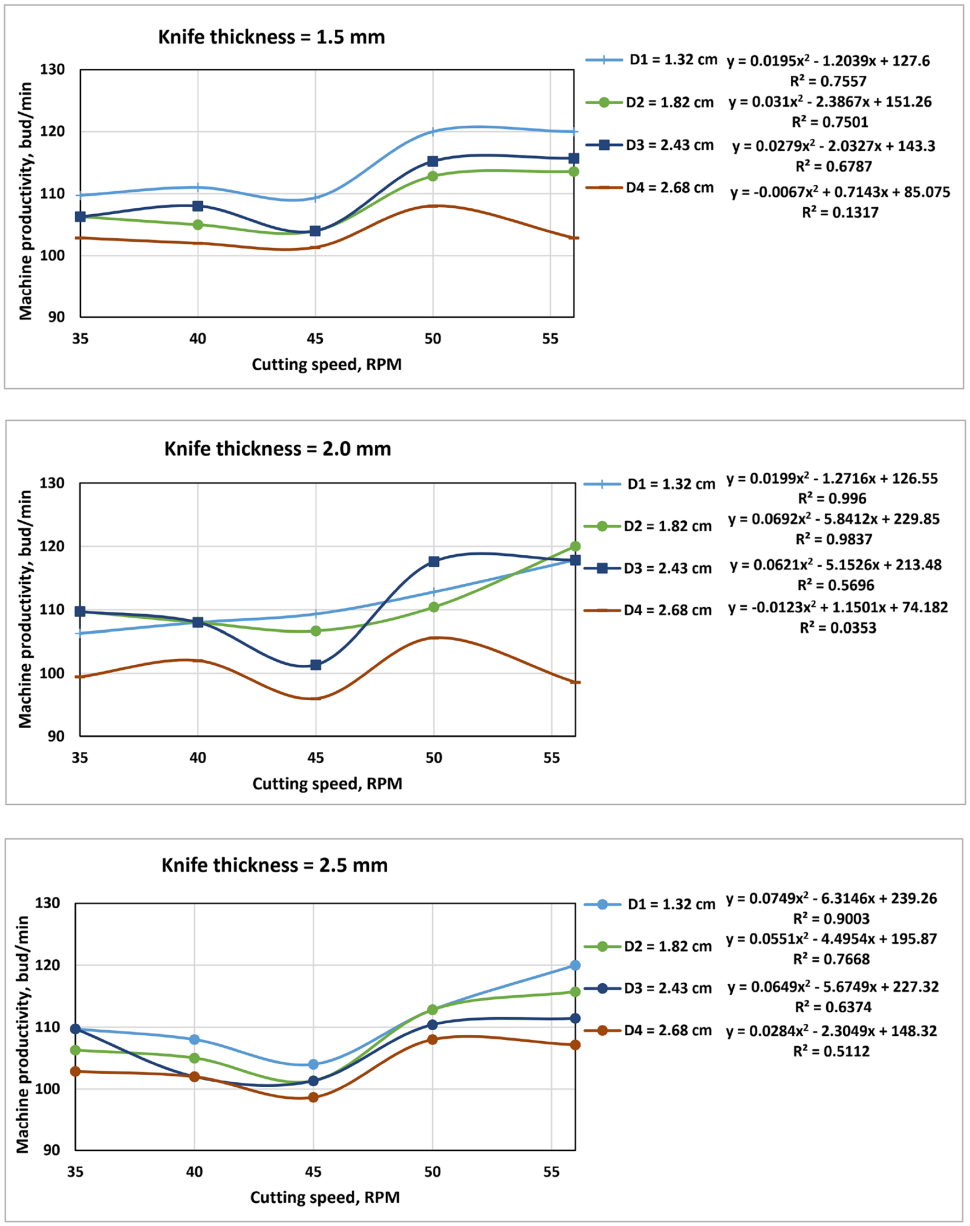
Machine productivity as a function of cutting speed.

The following figure (Fig. 13) shows the relationship between machine productivity and cutting speed, whereas the SSBC's greatest MP in the field was around 110 buds/min. It was also found that there is a direct relationship between the SSBC productivity and the speed of the cutting system and an inverse relationship between the SSBC productivity and the mean diameter of the SS. On the other hand, there are no significant differences in SSBC productivity as a result of changing the thickness of the cutting knife, as the results were very similar. These results agree with^4,8^.

Table 2 illustrates the analysis of variance (ANOVA) for the damage index, invisible losses, machine productivity, and skipping rate. Table 2 showed that there were significant effect between the treatment of each factors and also the interaction between them.

**Table 2:** Analysis of variance (ANOVA) for the damage index, invisible losses, machine productivity, and skipping rate.

S.V	d.f	M.S			
		Damage index	Invisible losses	Machine productivity	Skipping rate
A	2	44.511**	2886.279**	32.86*	22.200*
B	3	50.527**	589.517**	710.453**	506.151**
C	4	51.273**	2685.381**	708.417**	300.590**
A*B	6	43.968**	31.857	53.440*	39.042**
A*C	8	44.428*	55.0543*	15.246*	64.775**
A*B*C	36	43.640*	86.498**	21.236*	22.791**
Error	120	44.142	22.150	58.644	10.243

Where: Factor A = Knife thickness (1.5 mm, 2.0 mm, and 2.5 mm); Factor B = sugarcane stalk diameter (1.32 cm, 1.82 cm, 2.43 cm and 2.68 cm); Factor C = cutting speed (56 rpm, 50 rpm, 45 rpm, 40 rpm and 35 rpm); S.V. = Source of variance; d.f. = Degree of freedom; M.S. = Mean of squares; * Significant at P (0.05); ** Highly significant at P (0.01).

In addition, Table 3 shows the comparison among our work and the six references at the international level with SSBC. For examples Zhou et al.^32^ designed and testing a SBCM based on machine vision and reported that Machine Productivity (MP) was 40 buds/min However, detecting stem nodes quickly and accurately is still a significant challenge^33^. Ahmad et al.^23^ designed and testing a prototype of SBCM for nursery planting and reported that that MP was 18 bud/min. Mahmoud et al.^24^ developed a SBCM and reported that the maximum skipping rate, and MP was 11% and 36 bud/min. Researchers^17^ manufactured and evaluated the performance of a SBCM and reported that the MP was 66.7 bud/min. Wang et al.^34^ designed and evaluated a SBCM based on machine vision in pre-seed mode and reported that the average cutting time of a single seed is about 0.7 s (85 bud/min). Jadhav et al.^7^ designed and fabricated of a semiautomatic SBCM and reported that MP was 30 bud/min.

**Table 3:** Comparison of the previously different SBCM.

S. no	Res	Year	Machine type	Knife type	Design simplicity	Manufacturing Cost	Skills require for machine operating	Performance tests			
								Machine productivity	Skipping rate	Invisible losses	Damage index
1	^32^	2020	Based on machine vision	Rotary	Low	High	High	40	N.D.*	N.D	N.D
2	^23^	2020	Semiautomatic	Reciprocating	Medium	Medium	Low	18	N.D	N.D	N.D
3	^24^	2021	Semiautomatic	Reciprocating	Medium	Medium	Low	36	4.09–11%	N.D	N.D
4	^4^	2021	Semiautomatic	Reciprocating	Medium	Medium	Low	116	N.D	N.D	N.D
5	^34^	2022	Based on machine vision	Rotary	Low	Medium	Low	85	N.D	N.D	N.D
6	^7^	2023	Semiautomatic	Reciprocating	Medium	High	High	30	N.D	N.D	N.D
SSBC in this paper			Semiautomatic	Pivot	High	Medium	Low	110	0–13%	D**	D**

*Not detective, **Detected.

## Conclusions

A SSBC assisted with pivot knives was prototyped and proposed as an alternative against the manual method of cutting sugarcane stalks. The SSBC consists of a machine frame, cutting device and power supply source. The SSBC was experimentally evaluated for cutting sugarcane buds and was compared to the previously different SBCM. Field experiments were conducted to determine the damage index, invisible losses, machine productivity, and skipping rate as a function of SS diameter, cutting speed, and knife thickness. In the field tests five cutting speeds (35, 40, 45, 50, and 56 rpm), three cutting knives (1.5, 2.0, and 2.5 mm) were used for cutting sugarcane stalk with four different diameters (1.32, 1.82, 2.43, and 2.68 cm).

The main findings from the conducted study are presented below:

- DI values were less than 0.00 for all thicknesses of cutting knives, cutting speeds and stalk diameters under testing. It is inversely proportional to the cutting speed and knife thickness.
- The minimum invisible losses were recorded for the cutting knife thickness of 1.5 mm at a cutting speed of 56 r.p.m. and an average stalk diameter of 1.32 cm. It is directly proportional to the thickness of the cutting knives and inversely proportional to the cutting speed.
- The skipping rate was based mainly on the cutting speed, where it is directly proportional to the cutting speed, and we found that the highest skipping rate was recorded at the cutting speed 56 rpm.
- The productivity of the machine is directly proportional to the cutting speed and inversely proportional to the diameter of the cane sticks, while it does not depend on the thickness of the cutting knives.

Although the prototype can cut sugarcane cuttings precisely, with high production rate and less losses, it lacks automatic cutting detection. In the future, the machine will be developed to operate automatically while maintaining current specifications and simplicity of design. At the same time, technology will be used that is cheap, easy to operate and commercially viable.

## Data Availability

Data are presented in the article.

## List of symbols

$Q$: Machine productivity (bud/min)
*Nb*_A__*ctu*_: Actual cutting buds per time
*t*: Timed taken (min)
S_r_: Skipping rate (%)
${Nb}_{Theo}$: Theoretical cutting rate per time
*P*_*SD*_: Weight attributed (without damage)
*P*_*PD*_: Weight attributed (partial damage)
*P*_*ED*_: Weight attributed (extreme damage)
*n*_*SD*_: Number of buds (without damage)
*n*_*PD*_: Number of buds (partial damage)
*n*_*ED*_: Number of buds (without damage)
${L}_{i}$: Invisible losses (g per SS)
${W}_{i}$: Initial weight of SS before cutting (g)
${w}_{n}$: Weight of buds after cutting (g)
${N}_{n}$: Number of buds after cutting
${w}_{i}$: Weight of internodes after cutting (g)
${N}_{n}$: Number of internodes after cutting
${w}_{n}$: Weight of buds after cutting (g)
${N}_{n}$: Number of buds after cutting
${w}_{i}$: Weight of internodes after cutting (g)
${N}_{n}$: Number of internodes after cutting

## Abbreviations

SS: Sugarcane stalk
SBCM: Sugarcane bud cutting machine
MP: Machine productivity
SSBC: Semiautomatic sugarcane bud chipper
RFMU: Research farm at El-Minia University
ID: Damage index
SD: Without damage
PD: Partial damage
ED: Extreme damage
